# Cost-Effectiveness of Upper Extremity Dry Needling in Chronic Stroke

**DOI:** 10.3390/healthcare10010160

**Published:** 2022-01-14

**Authors:** Daniel Fernández-Sanchis, Natalia Brandín-de la Cruz, Carolina Jiménez-Sánchez, Marina Gil-Calvo, Pablo Herrero, Sandra Calvo

**Affiliations:** 1 Faculty of Health Sciences, Universidad San Jorge, Autovía A-23 Zaragoza-Huesca Km.299, Villanueva de Gállego, 50830 Zaragoza, Spain; efernandez@usj.es (D.F.-S.); nbrandin@usj.es (N.B.-d.l.C.); cjimenez@usj.es (C.J.-S.); 2 IIS Aragon, University of Zaragoza, Avda. San Juan Bosco, 13, 50009 Zaragoza, Spain; magilcal@unizar.es (M.G.-C.); sandracalvo@unizar.es (S.C.); 3 Department of Physiatry and Nursing, Faculty of Health Sciences, University of Zaragoza, C/Domingo Miral s/n., 50009 Zaragoza, Spain

**Keywords:** cost–utility, stroke, upper extremity, EQ-5D

## Abstract

Introduction: Dry needling is a non-pharmacological approach that has proven to be effective in different neurological conditions. Objective: The aim of this study was to evaluate the cost-effectiveness of a single dry needling session in patients with chronic stroke. Methods: A cost-effectiveness analysis was performed based on a randomized controlled clinical trial. The results obtained from the values of the EuroQol-5D questionnaire and the Modified Modified Ashworth Scale were processed in order to obtain the percentage of treatment responders and the quality-adjusted life years (QALYs) for each alternative. The cost analysis was that of the hospital, clinic, or health center, including the equipment and physiotherapist. The cost per respondent and the incremental cost-effectiveness ratio of each alternative were assessed. Results: Twenty-three patients with stroke were selected. The cost of DN treatment was EUR 14.96, and the data analysis showed a favorable cost-effectiveness ratio of both EUR/QALY and EUR/responder for IG, although the sensitivity analysis using limit values did not confirm the dominance (higher effectiveness with less cost) of the dry needling over the sham dry needling. Conclusions: Dry needling is an affordable alternative with good results in the cost-effectiveness analysis—both immediately, and after two weeks of treatment—compared to sham dry needling in persons with chronic stroke.

## 1. Introduction

Stroke is a major contributor to disability worldwide, and the second leading cause of death in Spain [1,2], generating a great impact on patients´ quality of life (QOL) due to the functional limitations that it entails [3]. According to the Global Burden of Disease Study (GBD), the socioeconomic burden of stroke has increased over time, although there has been a decrease in its prevalence [2]. Stroke imposes a high burden in terms of direct and indirect costs: on the one hand, indirect costs because of lost productivity due to patients’ long-term disability, restricted social functioning, and premature death, leading to a detriment to the patients’ quality of life; on the other hand, direct costs of care resulting from costs of health professionals, hospital services, medications, etc. [4].

Upper motor neuron lesions may result in long-term positive and/or negative symptoms [5], which usually lead to different degrees of upper extremity disability. Spasticity is one of the more common symptoms, leading to progressive functional limitation and decrease in quality of life [6]. The current scientific evidence shows the effectiveness of different interventions for the rehabilitation of upper extremity post-stroke, such as robotics [7], virtual reality [8], and different physical therapy protocols for functional improvement [9]. Physical therapy treatments can be combined with other pharmacological interventions and/or other medical treatments, such as antispastic drugs or botulinum toxin type A (BTX-A) infiltration [10]. Recently, non-pharmacological approaches have been used, such as dry needling (DN) of myofascial trigger points, which is increasingly used to treat neurological conditions such as stroke [11,12,13,14], Parkinson´s disease [15], and multiple sclerosis [16]. Although the reasons for the increase in non-pharmacological treatments such as DN are not clear, the following factors could be relevant: (1) from the patient´s perspective, the adverse effects of pharmacological treatments, or a shift to more patient-centered treatments, where patients are more involved in decision making on different treatment alternatives; and (2) from the professional and health system perspective, the high costs of pharmacological treatments such as BTX-A infiltration.

In relation to DN and BTX-A, BTX-A is the most potent neurotoxin known, and its paralytic effect is due to the blockade of neuromuscular transmission [17]. On the other hand, DN acts by mechanically impairing the sensory or motor components of nerve endings and dysfunctional motor endplates that contribute to the abnormal functioning of contractile elements [18]. Therefore, the main difference between the two would be the mechanism of action, as DN provokes a mechanical disruption, whereas BTX-A works via a chemical denervation [17]. DN is considered to be an effective and safe treatment to improve function and spasticity in stroke patients [13,14,19] when applied by an experienced physiotherapist [20]. Moreover, although DN may have some adverse effects—such as bruising, bleeding and pain—it does not have the other adverse effects that BTX-A can have, such as weakness, anatomic denervation, or long-term immune resistance [21]. However, when compared with BTX-A, DN has fewer long-lasting effects, which would involve including more treatment sessions [13].

To the authors’ knowledge, there has only been one study analyzing the cost-effectiveness of DN in neurological conditions [22]—specifically in stroke patients in the subacute phase—showing that the addition of four sessions of DN treatment for the upper extremity appears to be cost-effective. Cost-effectiveness analysis of DN treatment is necessary in order to determine the economic impact of adding or substituting treatments in the routine clinical practice of healthcare centers. Therefore, the main aim of this study was to analyze the cost-effectiveness of DN in patients with stroke in a chronic phase, using a cost–utility analysis in EUR per quality-adjusted life years (QALY), as well as analyzing the response to treatment based on the Modified Modified Ashworth Scale (MMAS) as the main outcome variable.

## 2. Materials and Methods

### 2.1. Study Design

An economic evaluation was performed following a previous randomized controlled trial (RCT) conducted at the Aragon Association of Stroke in Zaragoza (Spain). The study was approved by the Ethics Committee of Aragon (reference no. P116/0160), and registered at Clinicaltrials.gov no. NCT03546517 on June 2018.

### 2.2. Participants

The sample size estimation in the RCT was 23 participants [14]. Patients were enrolled in the study if they fulfilled the following criteria: (1) 40–90 years old, with hemiparesis from stroke of more than six months evolution; (2) ability to follow instructions and reply to questionnaires; and (3) hypertonia in at least one of the muscles of the upper extremity according to the MMAS score. The exclusion criteria were as follows: (1) grade 0 or 4 of hypertonia measured with the MMAS; (2) previous treatment with BTX-A or other treatments for hypertonia in the previous six months; (3) other neurodegenerative conditions; (4) fear of needles or contraindication to treatment with DN; and (5) cognitive decline (≤ 24 points on the Mini-Mental State Examination test). All participants provided signed informed consent before participation in the study.

### 2.3. Intervention Conditions

There were two groups: the intervention group (IG), and the sham group (SG). Patients were randomized with a 1:1 allocation ratio using an online research randomizer sequence generator (http://www.randomizer.org (accessed on 9 June 2018)). After randomization, the physiotherapist performed the treatment according to the assignments. The IG received a single-session treatment of DN with the DNHS^®^ technique, whereas the SG received a sham intervention, placing the needles superficially at the skin level and simulating the intervention in the same treatment context [14,23,24]. The DNHS^®^ technique is an adaptation of traditional DN techniques, with specific diagnostic and application criteria for neurological patients. One of the main differences is that muscles are needled in submaximal stretch, and progression with the treatment is based on spasticity release and not on pain recognition [14,25]. The muscles treated were the biceps brachii and brachialis, flexor digitorum superficialis and profundus, extensor digitorum, adductor pollicis, and triceps brachii. Diagnosis of myofascial trigger points in the muscles selected was performed with clinical criteria, which have proven to be valid and reliable [26].

### 2.4. Main Measures

The two variables used for the cost-effectiveness study were the values of the EuroQol-5D questionnaire and the MMAS.

#### 2.4.1. EuroQol-5D

The EQ-5D (EQ-5D-3L) is a recognized patient-reported outcome questionnaire including five dimensions (mobility, self-care, usual activities, pain/discomfort, and anxiety/depression) with three levels each (no problems, some problems, and extreme problems). The EQ-5D-3L questionnaire has a Cronbach’s alpha value of 0.928, and it has proven to be a reliable tool to measure QOL among stoke survivors [27,28].

#### 2.4.2. Modified Modified Ashworth Scale

The MMAS is a clinical scale used to assess hypertonia, and has been widely used despite its subjective component [29]; it scores the resistance to passive movement ranging from 0 (no increase in muscle tone) to 4 (rigidity in flexion or extension), and it has exhibited a good intra- (ICC = 0.748) and inter-rater (ICC = 0.781) reliability for assessing hypertonia in persons who have had a stroke [30]. Flexor and extensor muscles of the elbow and wrist are evaluated by assessing the resistance when the affected muscle group is passively stretched [31].

An improvement of at least one point in the MMAS is considered to be a clinically significant change [32]. The percentage of responders to treatment according to the MMAS has already been used in previous stroke cost-effectiveness studies in order to directly assess the impact of this type of intervention on affected muscles [22,32].

### 2.5. Costs

The evaluation of costs was carried out from the perspective of the hospital, clinic, or health center. From this point of view, only the direct healthcare costs associated with the DNHS^®^ technique were considered: the DN materials and the cost of the physiotherapist session. The materials used were gauze, disinfectant, and needles, and the cost of the session was determined based on public data from the official bulletins of five representative regions of Spain (Aragón [33], Castilla y León [34], Madrid [35], País Vasco [36], and Cataluña [37]). These bulletins publish the prices established for the provision of health services outside the public health system.

### 2.6. Outcomes

#### 2.6.1. Quality of Life (QOL)

QOL was measured at the beginning and two weeks after the DN intervention, and then the QALY was estimated using the area under the curve analysis. QALY is the preferred measure of health outcomes for use in technological appraisals, because it combines the impact of gains in QOL and in quantity of life (years) associated with an intervention [38]. The economic analysis through QOL was carried out by combining these data with the costs of the two interventions (DN and sham-DN), determining the incremental cost-effectiveness ratio (ICER). The ICER was calculated by dividing the difference in total costs by the difference in QOL, which represents the extra cost per extra unite of QOL [39]. The data were aggregated to the cost-effectiveness plane (graph), and were compared with the accepted cost-effectiveness threshold of EUR 20,000/QALY [40].

#### 2.6.2. Modified Modified Ashworth Scale (MMAS)

MMAS values were obtained before and after the first DN session, and again two weeks later. In order to be able to use these values in the economic analysis, it was necessary to transform this information into the number of responders to treatment. The number of responders was determined for each muscle, and the data were related to the costs of the intervention, indicating the cost per responder to treatment and the incremental cost-effectiveness ratio.

### 2.7. Statistical Analysis

Data were analyzed with SPSS version 25.0 (SPSS, Inc., Chicago, IL, USA), and were plotted with Excel (Microsoft, Redmond, WA, USA). Significant differences between quality-of-life measurements in the RCT [14] were confirmed, and the information was completed by McNemar’s test in the case of MMAS responders. To complete the sensitivity of the study, the minimum and maximum QOL values were added, and the data were compared to a cost-effectiveness threshold on a cost-effectiveness plane.

## 3. Results

A total of 23 patients aged 60.87 ± 15.16 years (mean ± SD; 61% male) were included in the final economic analysis; 11 were allocated to the IG and 12 to the SG, with no statistically significant differences between the groups at baseline.

### 3.1. Costs

The established costs of the treatment are displayed in Table 1. The DN materials used for one session cost EUR 0.64, and the mean physiotherapy cost per session according to published bulletins is EUR 14.32 ± 4.39. The DN material cost was the only difference between the IG and SG.

### 3.2. Quality of Life

**Figure 1:**
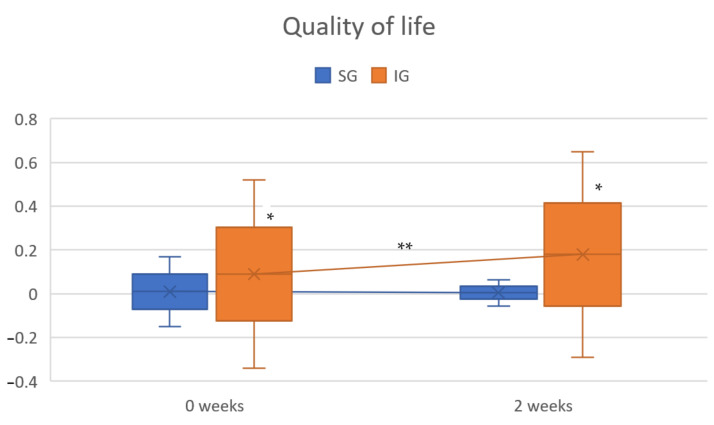
Variation of QOL during the study timeline. Abbreviations—IG: intervention group; SG: sham group. * *p* < 0.05 within IG; ** *p* < 0.05 between IG and SG.

The RCT results showed significant differences between groups in terms of QOL two weeks after the intervention (Table 2). The resulting QALYs were higher in the IG (Figure 1).

Costs and QALY are represented in a cost-effectiveness plane, with the accepted cost-effectiveness threshold of EUR 20,000 in red.

**Figure 2:**
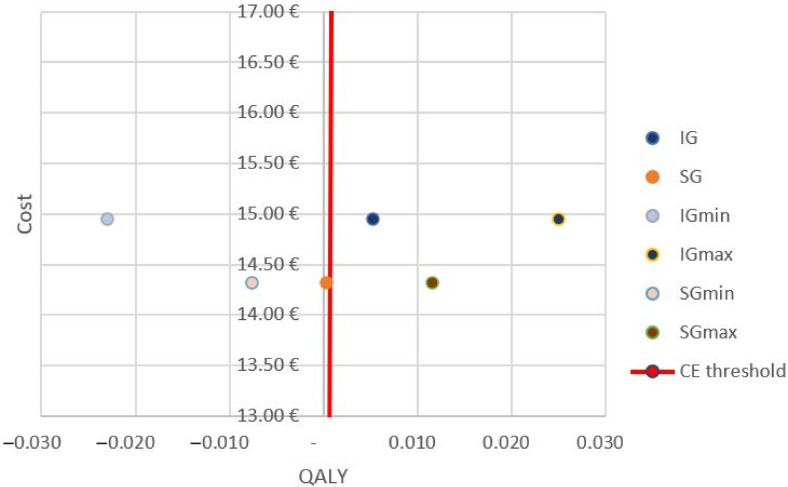
Cost-effectiveness plane. The average, minimum, and maximum values of the IG and SG are shown on the right-hand side of the cost-effectiveness threshold of EUR 20,000/QALY. Abbreviations—IG: intervention group; SG: sham group.

As can be seen in Figure 2, the values of IG, SGmax, and IGmax are below the cost-effectiveness threshold, which corresponds to the most favorable values for accepting the inclusion of this treatment. However, the values of SG, IGmin, and SGmin imply higher costs and/or lower QALY values.

### 3.3. Modified Modified Ashworth Scale

An improvement of one point on this scale in one of the five movements assessed was considered a response to treatment. In Table 3, it can be seen that the percentage of responders in the IG was higher in all of the muscles except for the elbow flexors. From the percentage of responders, it can be seen that the post-intervention cost per responder in the IG is on average almost half that of the control, and that at two weeks, the cost per responder remains the same in the IG, but the cost drops in the control (SG). However, the results of this scale only showed statistically significant improvements in the elbow extensors, with 73% of patients responding in the IG vs. 8% responding in the SG, considering the values taken before and just after the session with DN. The resulting ICER was low, indicating that DN only costs EUR 0.99 more than the alternative without DN to get one more respondent. The mean rate of treatment responders was higher in the IG than in the SG (39% vs. 20% after session and 41% vs. 28% two weeks after the treatment).

## 4. Discussion

This study explored the relationships between costs, QOL, and hypertonia when DN is applied to the upper extremity muscles of persons with chronic stroke, with the aim of analyzing the cost-effectiveness of a single DN session via a cost–utility analysis. Regarding costs, the main contributors when applying DN are the staff costs, since the materials used for DN have a very low cost. Although there are no fixed prices for the application of DN, and it may vary from country to country, in the case of Spain it can be calculated with relative certainty thanks to the official bulletins that publish the prices paid for services outside the Spanish public health system, as has been shown in previous studies [22,41].

Subsequently, the two variables we chose to assess the effectiveness of treatment were the QOL and the treatment response rate. The RCT results showed an improvement in QOL two weeks after the DN intervention, with significant differences between groups [14]. Other studies have also used the EQ-5D to assess the effectiveness of treatments in stroke patients but, unlike this study, they found no significant differences in quality of life between groups when applying DN [13] or BTX-A treatment [42]. It is possible that the limited number of patients included in the study may have influenced this, as the results were clearly better in the IG, with +0.0049 QALY compared to the SG.

On the other hand, we have a clinical variable, MMAS, which allows us to easily determine which patients are responders to treatment from a perspective other than that of the EQ-5D. In this case, significant differences were only obtained before and after treatment in elbow extensors, where we observed 65% more responders in the IG than in the SG. We remain unsure of the reasons for such a variety of responses to treatment, as well as why some muscle groups respond to treatment very differently than others, which is something worthy of consideration in future cost-effectiveness studies.

The results of the cost-effectiveness analysis indicate very low ICER values for both QOL and responder rate. The representation in Figure 2 allows us to see that the IG alternative is placed in the quadrant with the best cost-effectiveness ratio. However, the sensitivity analysis performed with the maximum and minimum values does not allow us to confirm the dominance (higher effectiveness with less cost) of the IG over the SG, since the minimum values of the IG could present worse QOL data, and the maximum values obtained for QOL in the SG show a cost-effective balance. In the economic analysis with the MMAS, the cost per respondent is EUR 41.54 less if valued just after the session, or EUR 17.54 less if valued two weeks after treatment. These results are consistent with another previously published study [22], and might suggest that adding DN to upper extremity rehabilitation treatment in stroke patients is a good alternative. The main reason for this is the low cost of this type of intervention which, combined with even a slight improvement in effectiveness variables, gives favorable results in the ICER. According to our data, to obtain one more QALY than the control group, we would only need to invest an additional EUR 130.14, and for responder patients the cost would be EUR 8.18 after treatment, or even a saving of EUR 0.83 at 2 weeks.

This study has certain limitations, such as the low number of patients evaluated, short follow-up period, and the use of only a single DN session. It is possible that a higher number of sessions may have a greater influence on QOL, as seen in the study of Cuenca Zaldívar et al., 2021 [13], where six needling sessions were performed. Moreover, the performance of only a single DN session limits the possibility of giving recommendations for the optimal number of sessions in terms of cost-effectiveness for the treatment of people with chronic stroke, which is something that could be done in the study carried out by Cuenca Zaldívar et al., who concluded that in the case of people with subacute stroke, four sessions of DN were more cost-effective than six sessions [13].

Since the first publication of the application of DN for spasticity in a child with cerebral palsy in 2007 [43], the number of studies of DN has increased and extended to many different neurological conditions, such as stroke [14], Parkinson’s disease [15], and multiple sclerosis [16]. However, although recent systematic reviews and meta-analyses have shown that DN is effective in decreasing spasticity, there has only been one study of cost-effectiveness in subacute stroke patients and, therefore, this study contributes to increase the evidence for the inclusion of DN treatment in rehabilitation protocols from the clinical and healthcare perspectives. This is important considering not only the effectiveness, but also the low costs compared to botulinum toxin, as the annual cost of the toxin vials for adult patients with spasticity in the upper extremity can range between EUR 529.87 and 1180.72 [44]. Moreover, from the patient’s perspective, it is important to consider that the treatment for focal spasticity is limited to BTX-A infiltrations [45] and, therefore, researching new treatment alternatives allows patients to empower themselves and seek treatments that are evidence-based, but under a patient-centered approach, considering their preferences [46,47].

Future lines of research should perform secondary analysis of RCTs of DN and other non-pharmacological interventions, including costs and cost-effectiveness. Apart from economic reasons derived from the costs of some pharmacological interventions, such as BTX-A infiltration, non-pharmacological treatments also have fewer adverse effects, and more research should be carried out from both the clinical and healthcare system perspectives, in order to analyze whether they can constitute an alternative to pharmacological treatments.

Although these results should be considered with caution due to the aforementioned limitations, the analysis performed shows that the inclusion of a single session of DN in the upper extremity rehabilitation protocols for chronic stroke patients can be of great benefit; therefore, future studies with a longer follow-up and a larger number of patients should be conducted in order to confirm these findings.

## 5. Conclusions

In this study, a cost-effectiveness analysis was conducted using two different effectiveness outcomes: the EQ-5D for QOL, and the treatment responders according to their hypertonia measured using the MMAS. The findings regarding the rate of responders showed good results in the cost-effectiveness analysis after treatment and at two weeks follow-up, finding that the application of DN in the upper extremity is an affordable alternative to use in patients with chronic stroke.

## Figures and Tables

**Table 1:** Cost of treatment.

	Unitary Cost	Sham Group	Intervention Group
Dry needling (material per session)	EUR 0.64	-	EUR 0.64
Mean physiotherapy cost per session	EUR 14.32 ± 4.39	EUR 14.32	EUR 14.96

**Table 2:** QOL, QALY, and ICER.

		Sham Group	Intervention Group
QOL	Pre-test	0.01 ± 0.16	0.09 ± 0.43 *
	2 weeks	0.005 ± 0.06	0.18 ± 0.47 *
QALY		0.0003 (min. −0.0077; max. 0.0115)	0.0052 (min. −0.0230, max. 0.02493)
ICER (EUR/QALY)		130.14 (min. −41.57, max. 47.51)	

* *p* < 0.05 within IG and between IG and SG.

**Table 3:** Rate of responders to treatment and cost per responder.

	Post-Intervention								2 Weeks							
	Control			Intervention (DNHS^®^)					Control			Intervention (DNHS^®^)				
	*n*	% Responder	EUR/Responder	*n*	% Responder	EUR/Responder	McNemar Test	ICER	*n*	% Responder	EUR/Responder	*n*	% Responder	EUR/Responder	McNemar Test	ICER
Elbow flexors	12	33%	EUR 42.96	11	27%	EUR 54.85	1.000	EUR −10.52	12	33%	EUR 42.96	11	27%	EUR 54.85	1.000	EUR −10.52
Elbow extensors	12	8%	EUR 171.84	11	73%	EUR 20.57	0.039 *	EUR 0.99	12	25%	EUR 57.28	11	73%	EUR 20.57	0.125	EUR 1.34
Wrist–dorsal flexors	12	25%	EUR 57.28	11	36%	EUR 41.13	1.000	EUR 5.61	12	17%	EUR 85.92	11	45%	EUR 32.91	0.375	EUR 2.22
Wrist–palmar flexors	12	17%	EUR 85.92	11	18%	EUR 82.27	1.000	EUR 42.09	12	17%	EUR 85.92	11	27%	EUR 54.85	1.000	EUR 6.01
Thumb adductor	12	17%	EUR 85.92	10	40%	EUR 37.39	0.375	EUR 2.73	12	50%	EUR 28.64	10	30%	EUR 49.86	0.625	EUR −3.19
Means		20%	EUR 88.78		39%	EUR 47.24		EUR 8.18		28%	EUR 60.14		41%	EUR 42.60		EUR −0.83

* *p* < 0.05 for elbow extensors post-intervention.

## Data Availability

Data are available on reasonable request by email to efernandez@usj.es.
